# Prevalence and Predictors of Poly-Victimization of Adolescents in England and Wales

**DOI:** 10.1177/08862605221118967

**Published:** 2022-08-29

**Authors:** Ferhat Tura, Eleftherios Nomikos, Lucy R. Betts

**Affiliations:** 1 Nottingham Trent University, UK

**Keywords:** adolescents, poly-victimization, risk factors, area deprivation, multilevel modeling

## Abstract

This study examined the change in the prevalence of adolescent poly-victimization and individual and area predictors of poly-victimization in England and Wales. The national representative longitudinal Offending, Crime and Justice Survey (2003–2006) was analyzed with data from 2,066 adolescents, aged between 10 and 18 years (mean ± *SD* at Time 1 = 13.08 ± 2.01), using multilevel multinomial logit models. Findings revealed that the majority of the adolescents (41.6%, 48.5%, 54.6%, 61.6%, respectively) did not experience victimization between 2003 and 2006. However, 28.3%, 25.9%, 19.5%, and 14.5% of the adolescents experienced poly-victimization (experiencing more than or equal to two types of victimizations), with a decrease of 13.8% over the 4-year period. Furthermore, some adolescents were consistent poly-victims, meaning they were poly-victims in all years that they participated in the survey. In particular, 3.57% of the adolescents who participated in the four waves of the survey were poly-victims in all years; 7.41% of the adolescents who participated in three of the four waves of the survey were poly-victims in all years; and 25.79% of the adolescents who participated in two of the four waves of the survey were poly-victims in both years. Statistically significant predictors of poly-victimization included having parents who have been in trouble with the police, offending, participating in community-related activities, being a boy, not managing income well, and living in an urban or deprived area. Offending had the greatest impact on poly-victimization. Findings highlight that adolescent poly-victimization in England and Wales decreased between 2003 and 2006 but some adolescents were more likely to experience poly-victimization due to individual, familial, and area characteristics. The findings therefore indicate that a holistic approach is needed to reduce adolescent poly-victimization and suggest that targeting area deprivation should be the priority.

Until recently researchers have tended to focus on adolescents’ offending patterns (Muncie, 2014). However, they are one of the most vulnerable groups in terms of experiencing crime as a victim (Jackson et al., 2017; Radford et al., 2013). Adolescents experience not only victimization types that non-vulnerable adults experience (e.g., burglary) but also specific forms of victimization (e.g., peer victimization, caregiver victimization). Further, the contexts where adolescents are victimized are also diverse (e.g., home, school, community, Martins et al., 2019). Despite these complexities of adolescent victimization, previous research in the United Kingdom has tended to focus on singular forms of victimization (e.g., DeCamp et al., 2018; Hayden & Dlugosz, 2012; Herlitz et al., 2016; Howard League, 2007; Roe & Ashe, 2008) rather than poly-victimization.

Poly-victimization is exposure to multiple forms of victimization and was originally defined as experiencing at least one victimization more than the mean number among the victim group as a whole (Finkelhor et al., 2005b, 2007), but there are various methods to construct poly-victimization, which are noted in the “poly-victimization” section later. Limited previous research on adolescent poly-victimization in the United Kingdom has explored the phenomenon at different geographical levels such as county (e.g., Jackson et al., 2016, 2017) and country (e.g., Fisher et al., 2015; Matthews et al., 2020; Radford et al., 2013, 2014), but previous research at the country level is still scarce and tends to use cross-sectional data (Fisher et al., 2015; Matthews et al., 2020; Radford et al., 2013, 2014). Many countries, including the United Kingdom, have experienced major drops in crime since the 1990s (Tseloni et al., 2010) and there is no previous research in the United Kingdom that explored the trends in poly-victimization of adolescents over time using longitudinal datasets. However, it is important to investigate the change in the prevalence of poly-victimization and the influence of time on the predictors measured (Caruana et al., 2015; Field, 2011) to identify those adolescents who are at most risk of experiencing poly-victimization repeatedly over time.

Furthermore, the number of predictors included in previous studies on adolescent poly-victimization in the United Kingdom are limited to sociodemographic variables such as gender, ethnicity, and family structure, whereas there are many other possible predictors of poly-victimization such as offending behavior, experiences with the police, frequency of going to a pub or club, and living in problematic social and area environments. For example, previous research found that those who offend (Turner et al., 2016), go to a pub or club frequently (Tseloni & Pease, 2015), and live in problematic social and area environments (Finkelhor et al., 2009; Sampson & Groves, 1989) are more likely to become a victim of crime.

Hence, the present study aimed to examine poly-victimization rather than singular forms of victimization and investigated the change in the prevalence of adolescent poly-victimization in England and Wales over time (Research Question 1) and identify individual and area predictors of adolescent poly-victimization (Research Question 2) drawing on the lifestyle and routine activities (L-RAT; Cohen & Felson, 1979; Hindelang et al., 1978), social bond (Hirschi, 1969), social disorganization (Sampson & Groves, 1989) theories, and the four pathways to poly-victimization framework of Finkelhor et al. (2009). For this, we ran multilevel multinomial logit models with MCMC (Markov chain Monte Carlo) estimation to explore the first and only national representative longitudinal survey (the Offending, Crime and Justice Survey (OCJS) from 2003 to 2006) that investigated the extent and nature of self-reported offending behavior, drug and alcohol use, attitudes and contact with the criminal justice system, and experiences of victimization of adolescents aged 10 to 18 in England and Wales (*N* = 2,066).

## Risk and Protective Factors of Poly-Victimization

Finkelhor et al. (2009) have identified four distinct pathways to poly-victimization: (1) residing in a dangerous community, (2) living in a dangerous family, (3) having a chaotic, multiproblem family environment, or (4) having emotional problems. However, testing these pathways in the UK context with secondary data analysis is not feasible. The secondary dataset used for the current study includes some indicators of these pathways, but they are not identical to the ones used in Finkelhor et al. (2009). In addition, these pathways consider only macro-level risk factors, whereas the current study also investigates individual risk and protective factors for poly-victimization. Therefore, we summarize the literature on the risk and protective factors of poly-victimization that are included in the survey used here drawing on the L-RAT, social bond, social disorganization theories, and where relevant the four pathways to poly-victimization framework of Finkelhor et al. (2009).

Previous studies have identified various risk and protective factors for adolescent victimization at the individual and area levels. Risk factors at the individual level are risky behaviors or lifestyles and routine activities of the individual, which are omitted in the four pathways to poly-victimization framework of Finkelhor et al. (2009). Risky behaviors include engaging in alcohol and drug use (DeCamp et al., 2018; Fagan & Mazerolle, 2011; Sampson & Lauritsen, 1990), which may lead to offending behavior (Jennings et al., 2012) that is associated with adolescent poly-victimization (Ford et al., 2010; Turner et al., 2016). Finkelhor et al. (2009) examine family drug or alcohol problems as part of the “having a chaotic, multiproblem family environment” pathway, but here the focus is on the individual’s alcohol and drug use, which might increase the likelihood of experiencing poly-victimization (Turner et al., 2016).

Previous research also demonstrates that in addition to risky behaviors like drug and alcohol use at the individual level, risky lifestyles and routine activities of individuals increase their risk of offending and being a victim of crime (Cohen & Felson, 1979; Finkelhor & Asdigian, 1996; Hindelang et al., 1978), which are omitted in the four pathways to poly-victimization framework of Finkelhor et al. (2009). The L-RAT argues that the convergence in time and space of a motivated offender, absence of a capable guardian, and presence of a suitable target generate criminal opportunities. In other words, those who engage in risky lifestyles (e.g., frequent club goers) are more likely to come in contact with motivated offenders in the absence of a guardian. For example, a UK-based study found that loneliness and poly-victimization were significantly associated (Matthews et al., 2020), which can be explained by the L-RAT (Cohen & Felson, 1979) as loneliness might indicate the absence of a capable guardian.

Social bond theory (Hirschi, 1969) has also been applied to study individual-level protective factors for adolescent victimization in the United Kingdom (e.g., DeCamp et al., 2018). Social bond theory argues that individuals are prevented from committing crime because of their social bonds. Specifically, more the individuals are securely attached to their parents and committed to their schools, the less likely they are to engage in offending behavior and be victimized. The theory also suggests that more the individuals are occupied with activities, the less likely they are to offend and be victimized. However, if the individuals have parents or friends who engage in problematic behavior, they are more likely to be involved in offending and be victimized (Esbensen et al., 1999; Finkelhor et al., 2009). For example, Lasky et al. (2021) found that participation in school activities increased the likelihood of poly-victimization, which can be explained by being away from home more frequently and therefore exposed to more motivated offenders.

At the area level, criminogenic environments with social disorganization (Finkelhor et al., 2009; Sampson & Groves, 1989) increase the risk of being a victim of crime (DeCamp et al., 2018; Finkelhor et al., 2009; Lasky et al., 2021). Previous research has suggested that community disadvantage (Kamndaya et al., 2017; Turner et al., 2016), or residing in a dangerous community (Finkelhor et al., 2009) influences the likelihood of experiencing poly-victimization.

Due to their established effects, previous research also controlled for several sociodemographic characteristics of individuals including age, gender, ethnicity, income, area type, and family structure (e.g., Turner et al., 2016), which were not included in the four-pathway framework of Finkelhor et al. (2009). Gender was found to be a significant predictor of poly-victimization, with boys being more likely to be poly-victims in the United States (Turner et al., 2016) and England and Wales (Radford et al., 2014). Adolescents living with two parents were underrepresented among poly-victims (Turner et al., 2016). Finally, while some studies found ethnicity as a protective factor (e.g., Lasky et al., 2021), in some studies it was not a significant predictor of poly-victimization (e.g., Jackson et al., 2016; Turner et al., 2016).

## Methodology

### Data

This study analyzed data from the longitudinal component of the OCJS, which was commissioned by the Home Office to investigate the extent and nature of self-reported offending behavior, drug and alcohol use, attitudes and contact with the criminal justice system, and experiences of victimization of those aged 10 to 25 living in private households in England and Wales. It is the first and only national representative longitudinal survey for both self-reported victimization experiences of adolescents and their offending behavior between 2003 and 2006 (Home Office 2008a, 2008b, 2008c, 2008d, 2009).

The OCJS had a “state-of-the-art” design for self-report offending surveys (Hales et al., 2009), and used a multistage stratified random sampling method. Respondents completed the questionnaires at home and parental consent was required for respondents aged 10 to 17. Only one adolescent was selected from each household. The final sample was national representative of the population aged 10 to 25 as the survey was designed as a “rotating panel.” That is, those who were interviewed in 2003, for example, were reinterviewed in 2004, 2005, or 2006, and a further “fresh” sample was introduced in 2004 onward to ensure a cross-sectional representative sample of young people aged 10 to 25, who were the focus of the longitudinal component of the survey (Home Office, 2008a).

In the present analysis, we included those who completed at least two waves of the survey (i.e., 2, 3, or 4) and who were aged between 10 and 18 in 2006 (*N* = 2,066) as we wanted to investigate adolescent poly-victimization. Of these adolescents, 364 participated in the 4 years of the survey; 323 in the first 3 years (2003**–**2005); 55 in 2003, 2004, and 2006; 60 in 2003, 2005, and 2006; and 318 in 2004, 2005, and 2006. Overall, 756 adolescents participated in three of the four survey waves. Of 2,066 adolescents, 209 participated in the first 2 years (2003**–**2004), 100 in 2003 and 2005, 27 in 2003 and 2006, 312 in 2004 and 2005, 59 in 2004 and 2006, and 239 in 2005 and 2006. Overall, 946 adolescents participated in 2 years of the four survey waves.

Based on the 2011 UK Census data (note that cross-tabulations—e.g., age by ethnicity are not possible with the 2001 Census data), our sample represents the 10- to 18-year-olds in England and Wales in terms of gender (range for the sample boys = 50.3%−52.1% vs. Census boys = 51.2%) and family structure (range for the sample one natural parent alone or with stepparent or other = 32.6%−33.6% vs. Census lone parent or stepfamily = 35.6%), but not ethnicity. While the percentage of White adolescents in our sample ranged from 90% to 90.9% (similar to that in Jackson et al., 2016), it was 81.7% in England and Wales (based on the 2011 UK Census data from White 10–17 years old adolescents).

### Poly-Victimization

Each year, the OCJS asked participants 10 questions about their victimization experiences in the last 12 months assessed as vehicle-related crimes (e.g., “Have you or anyone who lives here had their motor vehicles stolen or driven away without permission?”), burglary (e.g., “Has anything been stolen from your home, including from the garage, shed or garden?”), personal theft (e.g., “Has anyone stolen or tried to steal something that belonged to you?”), and violence (e.g., “Has anyone used force against you on purpose, for example, scratched, hit or kicked you, or used a weapon of any sort, or been violent to you in any way?”). Participants responded using a yes (1) or no (0) format. Initially, responses were summed and coded as a count variable (i.e., the total number of crime types experienced) to explore how many different types of crime respondents experienced in the past year for each year of the survey. That is, the 2003 wave measured victimization experiences in 2002, the 2004 wave in 2003, the 2005 wave in 2004, and the 2006 wave in 2005. Thereafter, we recoded this variable into three categories: zero victimization, one type of victimization, and two or more type of victimizations (i.e., poly-victimization) for each year. Consistent with previous research (e.g., Finkelhor et al., 2005b), and using the aforementioned count variable, we defined poly-victims as those who experienced one victimization more than the mean number among the victim group as a whole. In the current study this equated to two or more different victimization types as the average victimization was 1.08, 0.96, 0.74, and 0.60 in 2003, 2004, 2005, and 2006, respectively (see Table 1), which is in line with previous research that defined poly-victims as those who experienced more than one type of victimization (Bidarra et al., 2016; Turner et al., 2010). However, there are other methods to construct poly-victimization (see Segura et al., 2018) and a recent study (Lee et al., 2022) calls for a valid poly-victimization construct that is consistently agreed upon in the research community.

**Table 1.** Descriptive Statistics About Poly-Victimization and Predictor Variables.

Year		2003	2004	2005	2006
*N*		1,138	1,640	1,716	1,122
Poly-victimization	Categories	%			
Victimization	Zero	41.6	48.5	54.6	61.6
	One	30.1	25.7	25.9	23.9
	Poly-victim	28.3	25.9	19.5	14.5
	Mean	1.08	0.96	0.74	0.60
	Std. deviation	1.26	1.21	1.02	0.95
Predictor variables	Categories	%			
Alcohol use	No	96.9	98.8	99.7	99.8
	Yes	3.1	1.2	0.3	0.2
Drug use	No	93.3	90.4	89.0	88.6
	Yes	6.7	9.6	11.0	11.4
Pub visit	Never	74.3	65.8	62.0	57.1
	Less than once a week	21.5	27.4	30.4	33.9
	Once a week or more	4.2	6.8	7.6	9.0
Club visit	Never	81.0	74.5	69.8	66.8
	Less than once a week	17.7	23.0	27.6	31.4
	Once a week or more	1.3	2.5	2.6	1.9
Deviant parents	No	90.1	90.9	91.6	92.3
	Yes	9.9	9.1	8.4	7.7
Deviant siblings/friends	No	77.6	77.6	76.7	75.8
	Yes	22.4	22.4	23.3	24.2
Index of multiple deprivation (1–10)	Mean	5.37	5.30	5.22	4.72
	Std. Deviation	2.65	2.65	2.69	2.66
Offending	No	79.6	74.8	76.6	78.5
	Yes	20.4	25.2	23.4	21.5
School attachment	Like	23.5	25.1	25.2	22.2
	Don’t mind	52.5	52.0	52.3	54.5
	Don’t like	24.0	22.9	22.5	23.3
School-related activities	No	23.3	34.0	36.2	37.2
	Yes	76.7	66.0	63.8	62.8
Community-related activities	No	83.0	77.9	78.5	79.5
	Yes	17.0	22.1	21.5	20.5
Age	Mean	13.08	13.80	14.51	15.09
	Std. deviation	2.01	2.09	2.04	1.82
Predictor variables	Categories	%			
Gender	Boy	52.1	51.3	50.3	52.0
	Girl	47.9	48.7	49.7	48.0
Ethnicity	White	90.9	90.3	90.0	90.8
	Non-White	9.1	9.7	10.0	9.2
Income management	Managing well	62.1	65.9	63.5	66.0
	Getting by	33.5	30.7	32.9	30.6
	Getting into difficulties	4.4	3.5	3.6	3.5
Area type	Urban, >10K	80.2	79.1	80.2	75.6
	Town and fringe	10.5	10.6	10.2	13.3
	Village, hamlet, and isolated dwelling	9.3	10.3	9.6	11.1
Family structure	Both natural parents	66.7	67.4	66.7	66.4
	One natural parent alone or with stepparent or other	33.3	32.6	33.3	33.6
Household tenure	Owners	75.1	76.1	76.7	78.0
	Rental sector	24.9	23.9	23.3	22.0

### Predictor Variables

Selection of the predictors of poly-victimization was informed by the L-RAT, social bond, social disorganization theories, and the four pathways to poly-victimization framework of Finkelhor et al. (2009) and were categorized into three: (a) problematic behaviors or lifestyles (informed by the L-RAT theory), (b) social bonds (informed by the social bond theory), (c) problematic social and area environments (informed by social disorganization theory, and the four pathways to poly-victimization framework of Finkelhor et al., 2009). Following conventions established in previous research (e.g., Turner et al., 2016) demographic characteristics were also included.

Problematic behaviors included alcohol and drug usage. For alcohol usage, participants reported whether they felt drunk more than once a month in the last 12 months (yes or no). For drug usage, participants reported using a yes/no response format whether they took any illegal drugs (including glue, solvents, gas or aerosols, amyl nitrites, cannabis, amphetamines, ecstasy, LSD or magic mushrooms, cocaine, crack, heroin, methadone) in the last 12 months.

Offending was measured with a binary (yes or no) variable indicating whether the participant offended within the past year.

Routine activities included the reported frequency of visiting a pub or club in the last 12 months with three possible answers: never, less than once a week, or once a week or more. Social bond variables included school attachment and involvement, and community involvement. Whether a respondent likes or liked school indicated school attachment. Participants responded using a 3-point scale: not liking it, not really minding it, or liking it. Binary variables were created for school or community involvement. School involvement was assessed as whether a respondent took part in any of a series of school groups/clubs (e.g., drama, arts, music or singing groups, sports clubs, gyms, exercise or dance groups, computer clubs/groups, or other school clubs) in the last 12 months using a yes/no format. Involvement in community was assessed as whether a respondent took part in any of a series of community-related groups (e.g., religious groups, volunteer organizations, or social clubs) in the last 12 months using a yes/no format.

Problematic social and area environments were assessed as whether parents had ever been in trouble with the police (i.e., deviant parents: yes or no), closest friends or siblings were in trouble with the police in the last 12 months (i.e., deviant friends: yes or no), and neighborhood multiple deprivation index ranging from 1 (least deprived) to 10 (most deprived).

Demographic variables included age in years, gender (girl or boy), ethnicity (White or non-White), how a household managed on the total household income (managing well, getting by, or getting into difficulties), where a respondent lived (a city with a population more than 10,000 people, town/fringe, or village), and family structure indicating whether a participant was brought up by two “natural” parents, one “natural” parent alone or with stepparent, or other. We measured income with three categories due to the large number of missing cases for a 12-point ordinal scale variable ranging from the lowest category of under £2,500 to the highest category of £50,000 or more. For participants aged 16 or younger, the family structure variable represented current family structure. For participants aged 17 or older, it represented family structure when they were 10 to 16 years old. Finally, a binary variable was included to measure whether the participant lived in a house that was owned or rented. Descriptive statistics about all independent variables are presented in Table 1.

### Analytical Strategy

We ran a series of multilevel multinomial logit models with MCMC estimation (Browne, 2019; Snijders & Bosker, 1999) to identify risk and protective factors for adolescent poly-victimization. The dependent variable had three values: 0, 1, and 2 or more victimizations. For this reason, the statistical modeling used multinomial logit specification. Those who did not experience any victimization and those who experienced one victimization were the reference categories, respectively. We used multilevel modeling due to the longitudinal nature of the dataset which provided a two-level hierarchy (years [*N* = 4] nested within respondents [*N* = 2,066]). We used a MCMC method as suggested by Browne (2019) for multinomial logit models to avoid biased results. Finally, we included random intercepts and random slopes for the time variable in the models to allow for between-adolescent and within-adolescent variations, respectively, and to minimize residual dependency over time (i.e., autocorrelation, Cheng et al., 2010). In addition, we tested cross-level interactions between the year and time-invariant variables.

Using MLwiN Version 3.03 (Charlton et al., 2019), an example of the modeling process is as follows. We first ran a “base” model without any predictor variables. Second, we entered all predictor variables to the “base” model, which we named the “saturated” model. Third, we excluded nonsignificant variables at the 0.05 level from the “saturated” model and ran another model which we labelled as the “significant only” model. If a category of a nominal variable in the “saturated” model was significant at the 0.05 level, all categories for that variable were kept in the “significant only” model. In addition, if a variable was significant in one of the parts of the “saturated” model (e.g., non-victim vs. poly-victim) but not in the other (e.g., non-victim vs. one-off victim), we kept that variable in both parts of the “significant only” model.

## Results

With regards to the first research question, findings revealed that the majority of the sample (41.6%, 48.5%, 54.6%, and 61.6%, respectively) did not experience victimization over the 4-year period. Those who experienced one-off victimization made up 30.1%, 25.7%, 25.9%, and 23.9% of the sample, respectively, with a decrease of 6.2% over the 4-year period. Those who experienced poly-victimization (≥two types of victimization) made up 28.3%, 25.9%, 19.5%, and 14.5% of the sample, respectively. Overall, poly-victimization of adolescents decreased between 2003 and 2006 by 13.8% over the 4-year period.

Some adolescents experienced poly-victimization in more than 1 year. As noted above, 28.3% of the adolescents (*n* = 322) were poly-victims in 2003. Almost 40% of them (39.13%) were poly-victims in 2004, 24.22% in 2005, and 11.80% in 2006 as well. In 2004, 25.9% of the adolescents (*n* = 424) were poly-victims, 29.95% of them were poly-victims in 2005, and 12.26% in 2006 as well. In 2005, 19.5% of the adolescents (*n* = 334) were poly-victims, and 20.66% of them were poly-victims in 2006 as well (see Table 2).

**Table 2.** Descriptive Statistics for Poly-Victimization Over Time.

Poly-Victimization in More Than 1 year			
Year	2003	2003 and 2004	2003 and 2005
*N* (%)	322 (28.3)	126 (39.13)	78 (24.22)
Year	2004	2004 and 2005	2004 and 2006
*N* (%)	424 (25.9)	127 (29.95)	52 (12.26)
Year	2005	2005 and 2006	
*N* (%)	334 (19.5)	69 (20.66)	
Consistent Poly-Victimization Over Time			
Number of Years Participated	Sample Size (*N*)	Poly-Victim (*n*)	Poly-Victim (%)
Four years	364	13	3.57
Three years	756	56	7.41
2003, 2004, 2005	323	25	7.74
2003, 2004, 2006	55	10	18.18
2003, 2005, 2006	60	6	10
2004, 2005, 2006	318	15	7.72
Two years	946	244	25.79
2003, 2004	209	78	37.32
2003, 2005	100	10	10
2003, 2006	27	9	33.33
2004, 2005	312	74	23.72
2004, 2006	59	14	23.73
2005, 2006	239	35	14.64

Some adolescents were consistent poly-victims, meaning they were poly-victims in all years they participated in the survey: 3.57% of the adolescents who participated in the four waves of the survey were poly-victims in all years; 7.41% of the adolescents who participated in three of the four waves of the survey were poly-victims in the 3 years they participated. In particular, 7.74% of the adolescents who participated in 2003, 2004, and 2005 were poly-victims in all these years; 18.18% of the adolescents who participated in 2003, 2004, and 2006 were poly-victims in all these years; 10% of the adolescents who participated in 2003, 2005, and 2006 were poly-victims in all these years; and finally, 7.72% of the adolescents who participated in 2004, 2005, and 2006 were poly-victims in all these years (see Table 2).

Some adolescents participated in only two of the four waves of the survey and 25.79% of them were poly-victims in the 2 years they participated. In particular, 37.32% of the adolescents who participated in the survey in both 2003 and 2004 were poly-victims in both years; 10% of the adolescents who participated in the survey in both 2003 and 2005 were poly-victims in both years; 33.33% of the adolescents who participated in the survey in both 2003 and 2006 were poly-victims in both years; 23.72% of the adolescents who participated in the survey in both 2004 and 2005 were poly-victims in both years; 23.73% of the adolescents who participated in the survey in both 2004 and 2006 were poly-victims in both years; and finally 14.64% of the adolescents who participated in the survey in both 2005 and 2006 were poly-victims in both years (see Table 2).

The results, including the odds ratios, from the multilevel multinomial logit models predicting risk and protective factors for adolescent poly-victimization in England and Wales are presented in Table 3. Here we interpret the statistically significant results from the “significant only” models and the “saturated” models contain the nonsignificant results (see Supplemental Table 1).

**Table 3.** Multilevel Multinomial Logit Regression Models Predicting Risk Factors of Poly-Victimization.

Fixed Part	Sig. Only Model	
	β (SE)	OR (95% CI)
Non-victim versus poly-victim		
Intercept	−3.616 (0.265)***	0.03 (−0.49, 0.55)
Year (grand mean centered)	−.432 (0.064)***	0.65 (0.52, 0.77)
Felt drunk (Ref: No)		
Yes	1.101 (0.49)*	3.01 (2.05, 3.97)
Club going (Ref: Never)		
Less than once a week	.382 (0.135)**	1.47 (1.20, 1.73)
Once a week or more	1.12 (0.35)**	3.06 (2.38, 3.75)
Deviant parents (Ref: No)		
Yes	.736 (0.214)***	2.09 (1.67, 2.51)
Deviant friends (Ref: No)		
Yes	.463 (0.121)***	1.59 (1.35, 1.83)
Index of multiple deprivation (grand mean centered)	.083 (0.023)***	1.09 (1.04, 1.13)
Committed offense (Ref: No)		
Yes	1.606 (0.121)***	4.98 (4.75, 5.22)
School-related activities (Ref: No)		
Yes	.365 (0.115)**	1.44 (1.22, 1.67)
Community-related activities (Ref: No)		
Yes	.388 (0.131)**	1.47 (1.22, 1.73)
Age (grand mean centered)	−.102 (0.033)**	0.90 (0.84, 0.97)
Sex (Ref: Female)		
Male	.588 (0.126)***	1.80 (1.55, 2.05)
Income (Ref: Managing well)		
Getting by	.361 (0.116)**	1.43 (1.21, 1.66)
Getting into difficulties	1.005 (0.262)***	2.73 (2.22, 3.25)
Area type (Ref: Village, hamlet)		
Urban, 10K	.984 (0.232)***	2.68 (2.22, 3.13)
Town/fringe	.453 (0.286)	1.57 (1.01, 2.13)
One victim versus poly-victim		
Intercept	−1.877 (0.24)***	0.15 (−0.32, 0.62)
Year (grand mean centered)	−.185 (0.064)**	0.83 (0.71, 0.96)
Club going (Ref: Never)		
Less than once a week	.088 (0.12)	1.09 (0.86, 1.33)
Once a week or more	.335 (0.294)	1.40 (0.82, 1.97)
Deviant parents (Ref: No)		
Yes	.347 (0.167)*	1.41 (1.09, 1.74)
Deviant friends (Ref: No)		
Yes	.077 (0.106)	1.08 (0.87, 1.29)
Index of multiple deprivation (grand mean centered)	.035 (0.019)*	1.04 (1.00, 1.07)
Committed offense (Ref: No)		
Yes	.657 (0.104)***	1.93 (1.73, 2.13)
Community-related activities (Ref: No)		
Yes	.283 (0.117)**	1.33 (1.10, 1.56)
Age (grand mean centered)	−.03 (0.028)	0.97 (0.92, 1.03)
Sex (Ref: Female)		
Male	.25 (0.104)**	1.28 (1.08, 1.49)
Income (Ref: Managing well)		
Getting by	.26 (0.105)**	1.30 (1.09, 1.50)
Getting into difficulties	.551 (0.226)**	1.73 (1.29, 2.18)
Area type (Ref: Village, hamlet)		
Urban, 10Kk	.512 (0.201)**	1.67 (1.27, 2.06)
Town/fringe	.317 (0.248)	1.37 (0.89, 1.86)
Upbringing (Ref: Both parents)		
One natural parent	.245 (0.107)*	1.28 (1.07, 1.49)

*Note*. We measured ethnicity as White and non-White to make the models more parsimonious as preliminary analyses found that Mixed, Asian, Black, and “Other” adolescents did not differ in terms of poly-victimization experiences compared to White adolescents. OR = odds ratio; CI = 95% confidence interval.

* *p* < .05, **p < .01, ***p < .001 (two-tailed tests).

According to the results from the statistical models, poly-victimization declines with increasing year compared to both non-victimization and one-off victimization and younger adolescents are at greater risk of experiencing poly-victimization compared to a non-victim. Those who had parents who have ever been in trouble with the police, committed a crime, took part in community-related groups; were a boy, did not manage income well, and lived in an urban area with a population more than 10,000 or a deprived area were more likely to become a poly-victim compared to a non-victim or one-off victim. Club going, having a friend or sibling who have ever been in trouble with the police, and participating in school-related activities were associated with greater risk of becoming a poly-victim compared to a non-victim, but were not a significant predictor of poly-victimization compared to one-off victimization. On the contrary, being brought up by one “natural” parent was associated with greater risk of becoming a poly-victim compared to becoming a one-off victim. Overall, committing an offence, struggling with income, living in an urban area with a population more than 10,000, and having parents who have ever been in trouble with the police had the strongest influence on poly-victimization.

We finally examined cross-level interactions between the year and time-invariant variables. None of the interaction terms were statistically significant, meaning differences in time-invariant variables do not change with year.

## Discussion

This study examined the change in the prevalence of adolescent poly-victimization in England and Wales over time and identified individual and area risk and protective factors for adolescent poly-victimization drawing upon the L-RAT, social bond, social disorganization theories, and the four pathways to poly-victimization framework of Finkelhor et al. (2009).

It is difficult to make a direct comparison between the current study and previous studies in terms of the findings due to the differences in constructing the poly-victimization measures and variables included in the analyses. However, the current research results are in line with findings from Jackson et al. (2016) that reported that 23.4% of the sample aged 13 to 16 were poly-victims in the past year but not with findings from Radford et al. (2014) that reported that 37.8% of the sample aged 11 to 17 were poly-victims in the past year. The discrepancy in the findings seems to be due to the number of items that Radford et al. (2014) used to measure prevalence of poly-victimization.

With regards to results from the statistical models, while the results from the current analysis support the L-RAT, they contradict the social bond theory. In other words, those who go to a club and adolescents who participate in school- or community-related groups are more likely to experience poly-victimization. The contradictory finding regarding the effect of social activity participation on victimization might be due to exposure to more deviant friends. Interaction with deviant peers influences drinking and smoking, which increase the likelihood of becoming a poly-victim (Turner et al., 2016). In addition, this might indicate that more the adolescents are out of the house, the more they are exposed to opportunities for victimization as the L-RAT contends (Cohen & Felson, 1979).

Two problematic behaviors were examined as predictors of poly-victimization. Feeling drunk more than once a month increases the likelihood of experiencing poly-victimization. Committing an offence was also associated with greater risk of becoming a poly-victim. This is not a surprising finding, considering the broader adolescent victimization literature where early onset behaviors such as substance use and/or drinking were significant predictors of victimization as well as offending (Akers & Lee, 1999; Pinchevsky et al., 2014; Turner et al., 2016). Findings on the implication of problematic behavior should be carefully interpreted, as they can act as coping mechanisms stemming from the environment of the victim (Sinha, 2008; Wills & Filer, 1996). In this case, consideration is needed to support those victims who are part of such environments as there is intergenerational transmission of substance abuse and offending (Chassin et al., 1993; Farrington et al., 2009; Junger et al., 2013; Simons et al, 1988).

Among demographic predictors, findings revealed that younger adolescents were more likely to experience poly-victimization. Gender was significantly associated with poly-victimization, indicating that boys were more likely to experience poly-victimization than girls. Although, these findings support previous research (Lasky et al., 2021) we did not account for the types or seriousness of victimization. Past studies have found that girls are more likely to experience sexual victimization, and that sexual victimization experiences act as a gatekeeper to increased likelihood of poly-victimization and overall incident severity (Radford et al., 2013, 2017). Accounting for the type of victimization, as well as the source (i.e., domestic or external environments) may change our findings and should be explored in future research. Ethnicity, which was previously identified as a protective factor (Lasky et al., 2021), was not a significant predictor in our study, which is in line with previous studies from the United Kingdom (Jackson et al., 2016) and the United States (Turner et al., 2016).

Area type and deprivation were significant predictors of poly-victimization. While past research in the United Kingdom has not investigated deprivation indices, it has been noted that adolescent poly-victims tend to be residents of violent and severely deprived neighborhoods (Finkelhor et al., 2009, 2011b; Kamndaya et al., 2017). All previous risk factors might be affected by living in a deprived area, such as having deviant friends which might lead to offending and then being victimized as well as lack of parental supervision (Weijters et al., 2009). Broader victimization studies have identified links between area deprivation and victimization risk drawing on classical theories such as social disorganization (Tewksbury et al., 2010) and the “residing in a dangerous community” pathway to poly-victimization (Finkelhor et al., 2009), lending support to the potential lack of appropriate role models in highly deprived areas.

## Implications

Poverty, which we observed through income proxies, and area deprivation are vital risk factors of poly-victimization. In this case, potentially due to the lack of disposable income, the number of legitimate activities an adolescent can do may be limited. Boredom has previously been found to increase deviancy in adolescents (Malizia, 2018), which could have a domino effect in our previous top-risk factors, such as offending. However, it is important not to omit the established effects of area deprivation and disadvantage on the caretakers and surrounding environments of a child. Violent neighborhoods (Finkelhor et al., 2009, 2011a), the internalization of delinquency (Messer et al., 2006), and the lack of parental supervision (Weijters et al., 2009) are hazards created by socioeconomic inequalities. Various of the observed risk factors of poly-victimization seem to derive from social inequalities. Such findings have previously been established in a parental neglect and abuse context, where significant relationships were observed between deprivation indices and caregiver abuse (Coulton et al., 2007; Gillham et al., 1998). Furthermore, the lack of disposable income limits the number of legitimate activities, indicating financial and materialistic inequalities. In turn, knowing that deviancy in adults can stem from mental and materialistic strain (Agnew, 2011; Kaufman, 2009), parents may turn into problematic role models for adolescents, who follow them through with imitation, thus internalizing deviancy as the norm (Courtois & Gendron, 2017). Intra-family victimization should not be overlooked, with younger children previously identified at increased risk of caretaker and/or sibling victimization (Finkelhor et al., 2011b).

Perhaps addressing such issues on a parental level could be an alternative through interventions (Barkley & Robin, 2014). Adolescent victims residing in deprived areas, irrespective of their routine activities, are unequally victimized in comparison to their less deprived counterparts. Tackling socioeconomic inequalities can then lead, according to our findings, to an equitable risk of victimization by minimizing the social environmental risks. As such, community and council initiatives offering opportunities of nondeviant, pro-social activities to disadvantaged adolescents could be deemed as a worthy endeavor in aiding victimization and deviancy reduction. The above recommendation may sound contrary to our finding that school–club engagement increases likelihood of victimization, but these initiatives can be useful under capable guardians’ monitoring.

Further possible policy implications arise from our study, but it is important to acknowledge the data are from 15 years ago. First, the necessity in addressing problematic behaviors is vital. In particular, offending has the greatest impact on poly-victimization in our sample. Addressing offending should then be a priority in school and public settings, considering the fact that we could not account for those settings in our analyses. To this end, an educated guess that sibling and guardian deviancy/criminality are linked to the above is due. Consequently, attention must be paid to cases where adolescents are part of a family environment that increases their risk of deviancy (see Finkelhor et al., 2009). While deviant peers were also noted as a significant predictor of poly-victimization, interventions within this domain can lead to more harm than good; labeling, othering, and self-fulfilling prophecies to name a few. Arguably, deviant friends would almost certainly come from similarly high-risk environments which would in turn render them vulnerable, as seen in deviancy cohort studies (Fergusson et al., 2000). With poly-victimization literature indicating the impact on an adolescent’s future outcomes, risky family environment interventions would prove most fruitful according to our findings, especially if offending is linked to such environments, which support the findings from Finkelhor et al. (2009) in terms of living in dangerous family pathway to poly-victimization.

## Limitations of the Study

The present study has made important contributions to the adolescent victimization literature by investigating the change in the prevalence of poly-victimization and its predictors over time, rather than singular forms of victimization. However, there are inevitable limitations of the study due to the nature of the data used. First, the analysis was based on one randomly selected adolescent in private households and the OCJS did not cover crime against those not residing in private accommodation. That is, other adolescents in the same household or living in non-private accommodation may have had different experiences which are not captured in the OCJS. Second, as opposed to the Juvenile Victimization Survey (Finkelhor et al., 2005a), which has up to more than 40 screening questions for a comprehensive assessment of child victimization (five modules) including conventional crime, child maltreatment, peer and sibling victimization, sexual victimizations, and witnessing and indirect victimization, the OCJS has 10 questions. Future longitudinal studies need to extend the crime types examined to fully understand the change in the prevalence of and risk factors for poly-victimization in England and Wales over time. Third, several variables had large quantities of missing values, which led to their exclusion from our models. As such, datasets which would improve on this aspect would also aid in providing a more complete picture of the factors involved in adolescent poly-victimization. Fourth, although we used index of multiple deprivation, the study was not able to thoroughly explore risk factors in relation contexts where adolescents are victimized, which might increase the risk of blaming the victims. Fifth, measuring gender as a binary variable is a limitation of the dataset used and therefore the current study. Those who identify themselves as nonbinary experience various forms of victimization in various contexts (Sterzing et al., 2017; Wike et al., 2021), but we were not able to capture to what extent they experience poly-victimization particularly. Sixth, the data were not representative of the 10- to 18-year-olds in England and Wales in terms of ethnicity which may limit the generalizability of the findings. Future research needs to investigate poly-victimization experiences of ethnic-minority adolescents in England and Wales in detail.

Finally, the time frame of the OCJS was limited to 4 years and the data were from 16 to 19 years ago (2003–2006), which may limit the extent to which the results reflect adolescents of 2022. For example, Brennan (2021, p. 18) noted, “new methods of communication, changes in leisure activities and lower overall crime rates today and higher rates of alcohol consumption in the mid-2000’s are ways in which period and cohort effects may differ between the [10–18-year-olds populations of 2003–2006 and 2022].” That is, adolescents spend more time at home as they are online almost twice as long than a decade ago (UNICEF, 2020). Similarly, hundreds of clubs have closed in recent years (Connolly, 2015; figures from the Association of Licensed Multiple Retailers), and games, food, and gyms are becoming more popular than going to a club (Booth & Halliday, 2018). Therefore, due to the changes in leisure activities, adolescents’ experiences of some forms of crimes analyzed here might have changed, but experiences of other forms of crime that were not included here, such as cyber-enabled victimization, might have increased (Trompeter et al., 2022). Further, the pandemic (COVID-19) has affected the degree to which adolescents participate in school- and community-related activities, which were found to be correlated with poly-victimization in the current study, due to restrictions imposed by the central government. Therefore, the related findings of the current study might not be relevant for such a rare period, but the risk of victimization is probably similar as all the restrictions have been lifted. Nondrinking among young people has increased between 2005 and 2015 in England (Fat et al., 2018), which might suggest that the number of adolescents experiencing victimization due to feeling drunk might have changed, but it should be noted that more recent studies also found a relationship between drinking and poly-victimization and offending (Turner et al., 2016). Finally, the level of child poverty in recent years is similar to that in the mid-2000s (Stewart & Reader, 2021). Therefore, the lives of adolescents have not improved in general (UNICEF, 2020) and vulnerability and peer and family influence that this study investigated are likely to remain relevant (see also Brennan, 2021).

Despite the limitations and the datedness of the data used, it should be noted that the OCJS remains the most comprehensive and most recent national longitudinal survey of offending behavior in England and Wales (Brennan, 2021), and it is the only survey to investigate the change in the prevalence of and risk factors for poly-victimization over time. Further, the findings from the current study in relation to prevalence of poly-victimization are in line with the findings from a more recent cross-sectional study (Jackson et al., 2016), and there are recent studies that found a relationship between poly-victimization and risk factors examined in the current study (e.g., Turner et al., 2016). In addition, vulnerabilities of adolescents in England and Wales have not improved dramatically over time (UNICEF, 2020). Therefore, the OCJS is still a relevant dataset to be used (see also recent studies that analyze the OCJS such as Brennan, 2019, 2021; DeCamp et al., 2018; Walters, 2018, 2022). Importantly, none of the current studies on poly-victimization in England and Wales have tested as many risk factors available in the OCJS as the current study; therefore, this study is necessary to advance the scientific knowledge in relation to poly-victimization of adolescents in England and Wales.

## Conclusion

The present study investigated the change in the prevalence of poly-victimization over time and the predictors of poly-victimization in England and Wales. While findings in relation to the prevalence of poly-victimization are in line with some previous UK-based studies, they were not with some others, but overall adolescent poly-victimization decreased by 13.8% between 2003 and 2006. Some adolescents were consistent poly-victims, meaning they were poly-victims in all years they participated in the survey. Findings from the multilevel multinomial logit models revealed a variety of risk factors for poly-victimization, with offending having the greatest impact. The predictors that were included in the analysis as protective factors such as involvement in school- and community-related activities were found to be risk factors, which is indeed in line with previous research. However, we urge the reader to be cautious when interpreting the findings as our data have limitations. With these noted limitations in mind, we conclude that the major risk factors of poly-victimization stem from familial deviance which has in the past been linked to area deprivation and can have severe consequences on adolescents’ routine activities and lead them toward drinking, drug use, and offending that are associated with becoming a poly-victim. Therefore, we recommend targeting area deprivation primarily supported by interventions to mitigate risky family conditions.

## Supplemental Material

sj-docx-1-jiv-10.1177_08862605221118967 – Supplemental material for Prevalence and Predictors of Poly-Victimization of Adolescents in England and Wales Click here for additional data file. Supplemental material, sj-docx-1-jiv-10.1177_08862605221118967 for Prevalence and Predictors of Poly-Victimization of Adolescents in England and Wales by Ferhat Tura, Eleftherios Nomikos and Lucy R. Betts in Journal of Interpersonal Violence
