# Supplementary material for: Prevalence and Predictors of Poly-Victimization of Adolescents in England and Wales
Source: J Interpers Violence. 2022 Aug 29;38(5-6):4688–713. doi: 10.1177/08862605221118967 (PMC9900687; doi:10.1177/08862605221118967)
Supplement: sj-docx-1-jiv-10.1177_08862605221118967 – Supplemental material for Prevalence and Predictors of Poly-Victimization of Adolescents in England and Wales [file sj-docx-1-jiv-10.1177_08862605221118967.docx]

**Appendix Table 1**

*Multi-level multinomial logit regression models predicting risk factors of poly-victimisation*

| **Fixed Part** | **Saturated model** | | **Sig. only model** | |
| --- | --- | --- | --- | --- |
| **Non-victim vs poly-victim** | β (SE) | OR (95% CI) | β (SE) | OR (95% CI) |
| Intercept | -3.8 (0.296) *** | 0.02 (-0.56, 0.60) | -3.616 (0.265) *** | 0.03 (-0.49, 0.55) |
| Year (Grand mean centred) | -0.432 (0.066) *** | 0.65 (0.52, 0.78) | -0.432 (0.064) *** | 0.65 (0.52, 0.77) |
| Felt drunk (Ref: No) |  |  |  |  |
| Yes | 1.043 (0.486) * | 2.84 (1.89, 3.79) | 1.101 (0.49) * | 3.01 (2.05, 3.97) |
| Taken any drugs (Ref: No) |  |  |  |  |
| Yes | 0.233 (0.184) | 1.26 (0.90, 1.62) |  |  |
| Visiting Pub (Ref: Never) |  |  |  |  |
| Less than once a week | -0.059 (0.135) | 0.94 (0.68, 1.21) |  |  |
| Once a week or more | -0.206 (0.248) | 0.81 (0.33, 1.30) |  |  |
| Club going (Ref: Never) |  |  |  |  |
| Less than once a week | 0.393 (0.144) ** | 1.48 (1.20, 1.76) | 0.382 (0.135) ** | 1.47 (1.20, 1.73) |
| Once a week or more | 1.236 (0.381) ** | 3.44 (2.70, 4.19) | 1.12 (0.35) ** | 3.06 (2.38, 3.75) |
| Deviant parents (Ref: No) |  |  |  |  |
| Yes | 0.658 (0.214) ** | 1.93 (1.51, 2.35) | 0.736 (0.214) *** | 2.09 (1.67, 2.51) |
| Deviant friends (Ref: No) |  |  |  |  |
| Yes | 0.431 (0.121) *** | 1.54 (1.30, 1.78) | 0.463 (0.121) *** | 1.59 (1.35, 1.83) |
| Index of multiple deprivation (Grand mean centred) | 0.076 (0.024) ** | 1.08 (1.03, 1.13) | 0.083 (0.023) *** | 1.09 (1.04, 1.13) |
| Committed offense (Ref: No) |  |  |  |  |
| Yes | 1.573 (0.123) *** | 4.82 (4.58, 5.06) | 1.606 (0.121) *** | 4.98 (4.75, 5.22) |
| Like(d) school (Ref: Like(d)) |  |  |  |  |
| Don’t mind school | 0.152 (0.157) | 1.16 (0.86, 1.47) |  |  |
| Don’t like school | 0.206 (0.183) | 1.23 (0.87, 1.59) |  |  |
| School-related activities (Ref: No) |  |  |  |  |
| Yes | 0.384 (0.115) *** | 1.47 (1.24, 1.69) | 0.365 (0.115) ** | 1.44 (1.22, 1.67) |
| Community-related activities (Ref: No) |  |  |  |  |
| Yes | 0.408 (0.131) ** | 1.50 (1.25, 1.76) | 0.388 (0.131) ** | 1.47 (1.22, 1.73) |
| Age (Grand mean centred) | -0.095 (0.036) ** | 0.91 (0.84, 0.98) | -0.102 (0.033) ** | 0.90 (0.84, 0.97) |
| Sex (Ref: Female) |  |  |  |  |
| Male | 0.586 (0.126) *** | 1.80 (1.55, 2.04) | 0.588 (0.126) *** | 1.80 (1.55, 2.05) |
| Ethnicity (Ref: White) |  |  |  |  |
| Non-White | 0.012 (0.212) | 1.01 (0.60, 1.43) |  |  |
| Income (Ref: Managing well) |  |  |  |  |
| Getting by | 0.311 (0.119) ** | 1.36 (1.13, 1.60) | 0.361 (0.116) ** | 1.43 (1.21, 1.66) |
| Getting into difficulties | 0.916 (0.264) *** | 2.50 (1.98, 3.02) | 1.005 (0.262) *** | 2.73 (2.22, 3.25) |
| Area type (Ref: Village, Hamlet) |  |  |  |  |
| Urban 10k | 0.984 (0.234) *** | 2.68 (2.22, 3.13) | 0.984 (0.232) *** | 2.68 (2.22, 3.13) |
| Town/Fringe | 0.448 (0.284) | 1.57 (1.01, 2.12) | 0.453 (0.286) | 1.57 (1.01, 2.13) |
| Upbringing (Ref: Both parents) |  |  |  |  |
| One natural parent | 0.159 (0.129) | 1.17 (0.92, 1.43) |  |  |
| Tenure (Ref: Owner) |  |  |  |  |
| Rental sector | 0.095 (0.156) | 1.10 (0.79, 1.41) |  |  |
| **One-victim vs poly-victim** | β (SE) | OR (95% CI) | β (SE) | OR (95% CI) |
| Intercept | -1.97 (0.261) *** | 0.14 (-0.37, 0.65) | -1.877 (0.24) *** | 0.15 (-0.32, 0.62) |
| Year (Grand mean centred) | -0.205 (0.06) * | 0.81 (0.70, 0.93) | -0.185 (0.064) ** | 0.83 (0.71, 0.96) |
| Felt drunk (Ref: No) |  |  |  |  |
| Yes | 0.381 (0.364) | 1.46 (0.75, 2.18) |  |  |
| Taken any drugs (Ref: No) |  |  |  |  |
| Yes | 0.019 (0.159) | 1.02 (0.71, 1.33) |  |  |
| Visiting Pub (Ref: Never) |  |  |  |  |
| Less than once a week | -0.106 (0.122) | 0.90 (0.66, 1.14) |  |  |
| Once a week or more | -0.156 (0.225) | 0.86 (0.41, 1.30) |  |  |
| Club going (Ref: Never) |  |  |  |  |
| Less than once a week | 0.12 (0.129) | 1.13 (0.87, 1.38) | 0.088 (0.12) | 1.09 (0.86, 1.33) |
| Once a week or more | 0.4 (0.328) | 1.49 (0.85, 2.13) | 0.335 (0.294) | 1.40 (0.82, 1.97) |
| Deviant parents (Ref: No) |  |  |  |  |
| Yes | 0.342 (0.167) * | 1.41 (1.08, 1.74) | 0.347 (0.167) * | 1.41 (1.09, 1.74) |
| Deviant friends (Ref: No) |  |  |  |  |
| Yes | 0.052 (0.106) | 1.05 (0.85, 1.26) | 0.077 (0.106) | 1.08 (0.87, 1.29) |
| Index of multiple deprivation (Grand mean centred) | 0.035 (0.019) * | 1.04 (1.00, 1.07) | 0.035 (0.019) * | 1.04 (1.00, 1.07) |
| Committed offense (Ref: No) |  |  |  |  |
| Yes | 0.651 (0.105) *** | 1.92 (1.71, 2.12) | 0.657 (0.104) *** | 1.93 (1.73, 2.13) |
| Like(d) school (Ref: Like(d)) |  |  |  |  |
| Don’t mind school | 0.068 (0.128) | 1.07 (0.82, 1.32) |  |  |
| Don’t like school | 0.245 (0.151) | 1.28 (0.98, 1.57) |  |  |
| School-related activities (Ref: No) |  |  |  |  |
| Yes | 0.072 (0.106) | 1.07 (0.87, 1.28) | 0.061 (0.105) | 1.06 (0.86, 1.27) |
| Community-related activities (Ref: No) |  |  |  |  |
| Yes | 0.3 (0.116) ** | 1.35 (1.12, 1.58) | 0.283 (0.117) ** | 1.33 (1.10, 1.56) |
| Age (Grand mean centred) | -0.021 (0.03) | 0.98 (0.92, 1.04) | -0.03 (0.028) | 0.97 (0.92, 1.03) |
| Sex (Ref: Female) |  |  |  |  |
| Male | 0.255 (0.104) ** | 1.29 (1.09, 1.49) | 0.25 (0.104) ** | 1.28 (1.08, 1.49) |
| Ethnicity (Ref: White) |  |  |  |  |
| Non-White | -0.053 (0.17) | 0.95 (0.62, 1.28) |  |  |
| Income (Ref: Managing well) |  |  |  |  |
| Getting by | 0.263 (0.107) ** | 1.30 (1.09, 1.51) | 0.26 (0.105) ** | 1.30 (1.09, 1.50) |
| Getting into difficulties | 0.56 (0.23) ** | 1.75 (1.30, 2.20) | 0.551 (0.226) ** | 1.73 (1.29, 2.18) |
| Area type (Ref: Village, Hamlet) |  |  |  |  |
| Urban 10k | 0.519 (0.201) ** | 1.68 (1.29, 2.07) | 0.512 (0.201) ** | 1.67 (1.27, 2.06) |
| Town/Fringe | 0.328 (0.246) | 1.39 (0.91, 1.87) | 0.317 (0.248) | 1.37 (0.89, 1.86) |
| Upbringing (Ref: Both parents) |  |  |  |  |
| One natural parent | 0.239 (0.109) * | 1.27 (1.06, 1.48) | 0.245 (0.107) * | 1.28 (1.07, 1.49) |
| Tenure (Ref: Owner) |  |  |  |  |
| Rental sector | -0.023 (0.13) | 0.98 (0.72, 1.23) |  |  |
|  | **Non-victim vs poly-victim** | | **One-victim vs poly-victim** | |
| **Random Part** | **Saturated model** | **Sig. only model** | **Saturated model** | **Sig. only model** |
| Level: id_long | β (SE) | β (SE) | β (SE) | β (SE) |
| Var(cons.One) | 0.996 (0.156) | 0.966 (0.152) | 0.837 (0.132) | 0.911 (0.144) |
| Covar(cons.Poly-victim/cons.One) | 1.563 (0.2) | 1.623 (0.206) | -0.836 (0.106) | -0.795 (0.114) |
| Var(cons.Poly-victim) | 3.505 (0.366) | 3.547 (0.371) | 1.133 (0.189) | 1.136 (0.177) |
| Covar((year-gm).One/cons.One) | 0.084 (0.09) | 0.07 (0.083) | 0.039 (0.067) | 0.067 (0.08) |
| Covar((year-gm).One/cons.Poly-victim) | 0.169 (0.146) | 0.125 (0.138) | -0.028 (0.078) | -0.042 (0.089) |
| Var((year-gm).One) | 0.214 (0.079) | 0.208 (0.081) | 0.071 (0.071) | 0.155 (0.081) |
| Covar((year-gm).Poly-victim/cons.One) | -0.055 (0.083) | -0.071 (0.067) | 0.084 (0.068) | 0.143 (0.079) |
| Covar((year-gm).Poly-victim/cons.Poly-victim) | -0.101 (0.159) | -0.124 (0.123) | -0.078 (0.076) | -0.107 (0.084) |
| Covar((year-gm).Poly-victim/(year-gm).One) | 0.009 (0.029) | 0.03 (0.051) | 0.026 (0.042) | 0.061 (0.054) |
| Var((year-gm).Poly-victim) | 0.03 (0.027) | 0.062 (0.05) | 0.057 (0.05) | 0.108 (0.062) |
| Level: year_long |  |  |  |  |
| Level: resp_indicator |  |  |  |  |
| Var(bcons.1) | 1 (0) | 1 (0) | 1 (0) | 1 (0) |
| Units: id_long | 2,066 | 2,066 | 2,066 | 2,066 |
| Units: year_long | 5,616 | 5,616 | 5,616 | 5,616 |
| Units: resp_indicator | 11,232 | 11,232 | 11,232 | 11,232 |
| Estimation: | MCMC | MCMC | MCMC | MCMC |
| DIC: | 10,182.25 | 10,163.96 | 10,173.79 | 10,157.28 |
| pD: | 1,427.033 | 1,378.448 | 1,176.875 | 1,272.203 |

*Note.* Parameters denoted by ‘.One’ is ‘.Zero’ for the ‘one-victim vs poly-victim’ column. We measured ethnicity as White and non-White to make the models more parsimonious as preliminary analyses found that Mixed, Asian, Black, and ‘Other’ adolescents did not differ in terms of poly-victimisation experiences compared to White adolescents. **p* < .05, ***p* <.01, ****p* < .001 (two-tailed tests)
